# Better Person, Better Society: A Different Perspective on the Association Between Instrumental Religiosity, Interpersonal Empathy and Social Justice Values

**DOI:** 10.3390/bs16030331

**Published:** 2026-02-27

**Authors:** Marina Alexandra Tudoran, Alexandru Neagoe, Cosmin Goian, Theofild-Andrei Lazăr, Laurențiu Gabriel Țîru

**Affiliations:** 1 Faculty of Sociology and Social Work, West University of Timisoara, 300223 Timișoara, Romania; marina.tudoran89@e-uvt.ro (M.A.T.); alexandru.neagoe@e-uvt.ro (A.N.); cosmin.goian@e-uvt.ro (C.G.); theofild.lazar@e-uvt.ro (T.-A.L.); 2 National Institute of Research and Development for Electrochemistry and Condensed Matter, Dr. A. Paunescu Podeanu Street No. 144, 300569 Timișoara, Romania

**Keywords:** social justice values, instrumental religiosity, interpersonal empathy, path analysis, mediation

## Abstract

Religiosity and empathy have been identified as two key variables that may significantly influence an individual’s social justice attitude and behavior. Despite their significance, studies addressing the relationships between these variables are rare. Thus, the present study aims to explore the associations between interpersonal empathy, instrumental religiosity, and social justice values using the conceptual framework of motivated information processing theory. Structural equation modeling (SEM) was employed to assess the hypothetical relationships between these variables. The findings indicate that personal instrumental religiosity, social interaction, and cognitive behavior are positively associated with the level of adherence to both instrumental and social terminal values of social justice. In contrast, social instrumental religiosity exerts only a direct influence on the instrumental values of social justice. This study also revealed the role of social interaction and cognitive behavior as mediators between personal instrumental religiosity and the instrumental and social terminal values of social justice. The findings underscore the imperative for researchers to devise educational programs that acknowledge and promote the significance of religion and empathy in fostering a more equitable and compassionate society.

## 1. Introduction

In the academic literature, the concept of justice is addressed through two main approaches. The predominant strategy within the fields of psychology and sociology is the descriptive method, which involves the examination of prevalent societal perspectives with the aim of elucidating the origins of resources and the manner in which they are distributed across diverse social groups. According to this perspective, it is argued that justice is a social construct that is unable to be universally applied and is inherently dependent on cultural and temporal influences. At the opposite end of the spectrum, the most popular approach among philosophers is the normative one, which attempts to reason about the nature of justice by proposing and evaluating various rational arguments. From a descriptive perspective, the constant pursuit of the true meaning of justice has not yielded the anticipated outcomes, as there appears to be no inherent or absolute justice beyond the socially constructed interpretations. From a normative perspective, empirical ethics is not compatible with the nature of moral judgments, which are rationally contestable, on the grounds that it assumes the correctness of social consensus and the incorrectness of the views of minorities and social reformers. This approach stands in opposition to the established practice of moral argumentation, resulting in a complete absence of rational contestation concerning moral disagreements (Gaertner & Schokkaert, 2012). Consequently, there has been a notable increase in empirical social research studies in recent decades, a necessity when seeking to apply the theory of justice in the real world. For instance, empirical research on the acceptance of notions of justice by different social groups plays a significant role in understanding the social environment in which political decisions are made. Furthermore, research findings have indicated a correlation between individuals’ personal beliefs regarding justice and specific personal attributes. In certain cases, empirical results can serve as a necessary complement to theoretical work, particularly in scenarios where ethical theory itself stipulates a requirement for empirical evidence. Alternatively, they may be essential in circumstances where a compromise must be made between conflicting axioms or conclusions (Gaertner & Schokkaert, 2012).

### 1.1. Motivated Information Processing Theory

In the literature, the theory that human knowledge is a social product is well known and accepted, with social influence concurrently being an inevitable part of human interaction. However, according to Kelley and Thibaut’s theory (Kelley & Thibaut, 1978), a better understanding of human behavior in interdependent situations requires consideration of the structure of the situation in which the interaction takes place; it has been found that, broadly speaking, any social behavior can be explained in terms of information processing (Forgas, 1983; De Dreu & Carnevale, 2003). The theory of motivated information processing is based on the assumption that individuals choose what to consider and how to respond to the information they receive (Anderson & Harbridge, 2014). In this context, De Dreu and Carnevale (2003) advanced a theoretical framework stating that actions and judgments in interpersonal scenarios are contingent on the interplay between epistemic and social motivations, with each type fulfilling a distinct function (De Dreu et al., 2008a; Shteynberg et al., 2017; Super et al., 2016). However, from the authors’ perspective, epistemic and social motivation were conceptualized as orthogonal constructs; the authors mention that there are situations in which both types can be influenced by certain antecedent conditions (De Dreu et al., 2008b). Thus, both types of motivation capture the influence of structural variables (e.g., cultural values, responsibility, ambient noise, and time pressure) and personality variables (e.g., need for knowledge, need for affiliation, agreeableness, and openness to experience). Within the framework of motivated information processing theory, individual epistemic motivation determines the extent to which new information is sought and generated and how deeply and deliberately this information is processed, while individual social motivation (pro-self vs. prosocial) influences what type of information is sought, generated, and processed. Thus, if high epistemic motivation leads to greater attention to the processing and integration of available information in order to form an opinion, social motivation will determine what type of information is relevant to that opinion (De Dreu et al., 2008b; Shteynberg et al., 2017). The interaction between social and epistemic motives has been studied in various fields, with the theory of motivated information processing finding application in explaining the behavior of negotiators, analyzing the learning process within a team, elucidating how conflict is resolved within a team, or studying the creativity of group members (Shteynberg et al., 2017).

### 1.2. Social Justice from the Religion Perspective

The relationship between religion and social justice has been shown to be profound and complex. In general terms, the moral frameworks espoused by religious teachings align with the principles of social justice, with a particular emphasis on fairness, equality, and the protection of human dignity. However, significant variations have been observed in the manner in which religion interacts with social justice, depending on the religious tradition, cultural context, and historical period. In certain instances, religious institutions have been instrumental in promoting social justice. Conversely, in other scenarios, religion has been employed to justify or perpetuate injustice against specific social groups (Liu, 2025). The link between social values and religion was re-evaluated in the 1950s and 1960s by Gordon Allport, who explained the contradictory effects of religion on social values by distinguishing between two fundamentally different religious orientations and motivations, namely intrinsic religious orientations and extrinsic religious orientations (Allport, 1966; Rogobete et al., 2021). According to Allport’s theory, the distinction between extrinsic and intrinsic religiosity differentiates believers who attend church but whose community membership supports and serves other (non-religious) purposes from believers for whom religion is an end in itself and a final good, not an instrumental one (Rogobete et al., 2021). Thus, intrinsic religiosity is indicative of the fundamental spirit of religious tradition reflected in a person’s predominant motivations, suggesting a commitment to the inherent principles of religion and involvement in religious services (Tariq et al., 2019). Individuals with an intrinsic religious orientation internalize religious values and practice religion for their own sake (Allport & Ross, 1967; Hall et al., 2008; Banazadeh et al., 2019). At the opposite end of the spectrum, extrinsic religiosity associates religion with an instrumental means of consolation and sociability, most often involving membership in a religion, social activities, and/or religious preferences (Foong et al., 2023). Although individuals with an extrinsic orientation have religious beliefs, for them, religion is a tool used to satisfy basic needs. In other words, this type of religiosity refers to those objective, real, and measurable behaviors that stem from religious beliefs and practices (Allport & Ross, 1967; Banazadeh et al., 2019). Despite the fact that this conceptualization has shaped most subsequent empirical research in the psycho-sociology of religion, the distinction between intrinsic and extrinsic religiosity has remained a controversial topic for contemporary researchers (Kirkpatrick & Hood, 1990; Hall et al., 2008).

### 1.3. Social Justice from the Empathy Perspective

The experience of perceiving another person’s injustice is a widespread human phenomenon in contemporary society (Shteynberg et al., 2017). In this context, empathy is considered to be the ability to feel what another person feels so that the emotional connection created favors prosocial behaviors towards that person (Suazo et al., 2020). In addition to recognizing the emotions and needs of others, empathy also requires experiencing emotional resonance and motivational transformation, all of which encourage proactive efforts to help others (Peng et al., 2024). Thus, empathy is considered a fundamental component of the motivational factors underlying care for others and the process of guiding moral judgments in various forms. It has also been found that empathy exerts a significant influence on a multitude of aspects of social interactions, as it is an essential component in decision-making processes (Decety & Cowell, 2015). Other studies have shown that the manner in which individuals formulate judgments concerning social justice is unique to each person and is influenced by empathy, given its impact on the decision-making process (Urbanska et al., 2019). However, the relationship between empathy and morality is context-dependent, and further research is needed to determine the extent to which these two characteristics are interconnected and how empathy influences people’s perceptions of fairness in contexts of social inequality (Decety & Cowell, 2015; Urbanska et al., 2019).

### 1.4. From Social Values to Social Justice

Values are defined as goals and motivations that serve as guiding principles in life, as they are considered a part of self-presentation, with the role of informing others about the quality of the individual. They are associated with identity and self-esteem and represent the driving force that influences evaluation, choices, and behavior (Feather, 1992; Bilsky & Schwartz, 1994; Juujärvi et al., 2012). Social values and moral norms can also be considered an important part of a society’s culture due to their functional role in the stability of social order while also providing general guidelines for social conduct and cooperation. Thus, people are motivated by social values that, once internalized morally, can inspire political participation and the courage to fight against injustice, regardless of personal costs (Yoder & Decety, 2022). In this context, moral decision-making is a conscious, effortful, and controllable process, but one that is influenced by emotions, involving the evaluation of actions in relation to the norms and values established in a social environment (Reniers et al., 2012). Following the same theoretical line, according to the motivated information processing theory, the thoughtful processing of social information is not an instantaneous phenomenon; rather, it is influenced by an individual’s motivation to carefully consider their own judgments and actions (Van Knippenberg et al., 2021). The literature also notes that perceptions of justice are shaped by personal experiences and social interpretation processes. However, although the experience of perceiving injustice in others is a widespread human phenomenon, there are still a limited number of studies on how justice perceived in others can become relevant to oneself. Research on the social construction of justice has predominantly concentrated on the immediate situational factors that facilitate justice transmission. For example, the social transmission of procedural justice has been studied by Shteynberg et al. (2017). Using the principles of motivated information processing theory, the authors explored how individual differences in social and epistemic motives determine specific evaluative and behavioral responses of individuals to the fair and unfair treatment of others. The authors state that although motivated information processing theory provides a useful conceptual framework for examining how the treatment of others influences an individual’s judgments, intentions, and behavior in matters of justice, it is possible that the interaction between social and epistemic motives also leads to the social transmission of other forms of justice, such as distributive and interpersonal justice (Shteynberg et al., 2017).

In this context, the objective of this study is to explore the relationship between instrumental religiosity, interpersonal empathy, and social justice values. Motivated information processing theory assumes that social and epistemic motivation together determine decisions, judgments, and information processing, and it was used as a model for analysis in this study. In addition, studies have confirmed that a person whose primary motivation is religion will internalize and follow their religion and its principles at any cost (Allport & Ross, 1967). According to this approach, a person will assume certain values associated with social justice either directly, due to the influence of the religious component, or indirectly, through the influence manifested on empathy.

## 2. Materials and Methods

### 2.1. Purpose of the Study and Hypotheses

The relationship between empathy, religion, and social justice has been comprehensively documented in the relevant literature. Torres-Harding et al. (2013) demonstrated that a positive correlation exists between an individual’s degree of religious devotion and their declared intentions to participate in activities pertaining to social justice (β = 0.31, *p* = 0.000, and R^2^ = 0.14). The correlation between attitudes and perceptions of social justice and individual empathy was confirmed by Cartabuke et al. (2019), who observed that students exhibiting high levels of empathy reported greater awareness of social justice issues (β = 0.19 and *p* < 0.01). Batson et al. (1993) analyzed the extent to which religion, similar to empathy, promotes altruistic behavior. Their findings indicate that certain expressions of religious behavior contribute to the promotion of prosocial behavior (Paciotti et al., 2011). Al Ghanam’s (2024) examined the interplay between religion, empathy and prosocial behavior and concluded that both variables significantly influence prosocial behavior, directly (β = 0.20 and *p* < 0.01; β = 0.17 and *p* < 0.01 respectively) and indirectly, through the influence of peer relationships. It has furthermore been confirmed in other studies that the mediating effect of empathy is present in a socio-religious context. As Ma and Jiang (2020) showed, empathy mediates the relationship between post-transgression benevolence motivations and forgiveness (β = 0.08 and CI: (0.05, 0.13)). In addition, Jiang et al. (2025) demonstrated that empathy tends to mediate the effect of spiritual health on spiritual care capacity, but with a very weak effect size (β = 0.019, SE = 0.008, and 95% CI = 0.003–0.035). However, to the best of the present author’s knowledge, no studies that examine the relationship between instrumental religiosity, interpersonal empathy, and social justice values have yet been identified in the literature.

Based on these considerations, this research aims to explore how instrumental religiosity and interpersonal empathy influence the assumption of values associated with social justice using the motivated information processing theory. According to this approach, the level of adherence to social justice values is influenced by the cumulative effect of epistemic (instrumental religiosity) and social (interpersonal empathy) motivations. The following hypotheses were formulated regarding the hypothetical relationships between the religious components, the empathic components, and social justice values:

**Hypothesis** **1a.** *There is a significant association between personal and social instrumental religiosity, the social interaction and cognitive behavior associated with interpersonal empathy, and instrumental values of social justice.*

**Hypothesis** **1b.** *The social interaction and cognitive behavior associated with interpersonal empathy mediate the relationship between personal and social instrumental religiosity and instrumental values of social justice.*

**Hypothesis** **2a.** *There is a significant association between personal and social instrumental religiosity, the social interaction and cognitive behavior associated with interpersonal empathy, and social terminal values of social justice.*

**Hypothesis** **2b.** *The social interaction and cognitive behavior associated with interpersonal empathy mediate the relationship between personal and social instrumental religiosity and social terminal values of social justice.*

**Figure 1:**
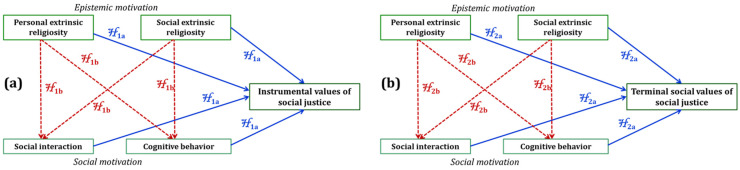
Schematic models used in this study. Dependent variables: (**a**) Instrumental values of social justice; (**b**) Terminal social values of social justice.

The conceptual diagram of this study is presented in Figure 1, showing the hypothesized relationships between the variables. The simple model is illustrated by the “a” symbol and the straight lines, while the mediated model is illustrated by the “b” symbol and the dashed lines.

### 2.2. Participants

In order to confirm the hypotheses, a cross-sectional study was conducted using self-reported data, focusing on students in western Romania from the social sciences departments of the universities of Reșița, Timișoara, Arad, Oradea, and Cluj-Napoca. Participation in the survey was voluntary, and no financial or academic incentives were offered to encourage participation. Participants had the opportunity to complete the questions at home, without classroom constraints, teacher influence, or the academic pressure associated with traditional educational settings. The survey was administered online and employed a forced-response format, wherein participants were required to respond to each item prior to advancing to the subsequent one. The average response time was 8 min, with a completion rate of 73.3%. The data were collected between March and June 2025. The sample consisted of 422 respondents who were selected using the convenience sampling method. In the absence of universally accepted criteria for determining the sample size based on the variables analyzed, the present study followed methodological recommendations from the existing literature (Gray et al., 2017; Sang et al., 2022). A sensitivity power analysis was also conducted prior to data analysis using G*Power 3.1.9.7. For the multiple regression corresponding to the proposed path model with four predictors, assuming an alpha level of 0.05 and a statistical power of 0.80, the analysis indicated a minimum detectable effect size of f^2^ = 0.029, which corresponds to a small effect (Faul et al., 2007). This corresponds to R^2^ = f^2^/(1 + f^2^) = 0.028 (i.e., the model would be powered to detect approximately 2.8% of the explained variance), which is equivalent to a multiple correlation of R = √R^2^ = 0.168. Table 1 presents the demographic data of the respondents.

### 2.3. Measurements

The current study employed a questionnaire developed in a previous study (Tudoran et al., 2024) to ascertain the extent to which respondents align with values associated with social justice. The other variables are self-assessment tools that were adapted and validated for the Romanian population. This process involved a repeated forward–backward translation procedure, which is the most common method in the adaptation and translation process (Mimura & Griffiths, 2004).

Social justice values were assessed using the social justice value scale (Tudoran et al., 2024). The scale comprises two dimensions: instrumental values of social justice, which define how individuals subjectively perceive the most appropriate cognitive and behavioral modes that respect the principles of social justice and terminal values of social justice, which establish the direction of value orientations that motivate individuals to engage in behaviors associated with social justice. Terminal values are further differentiated into social terminal values (socially oriented) and personal terminal values (self-oriented). A Cronbach’s α of 0.851 was obtained for instrumental values, 0.792 for social terminal values, and 0.732 for personal terminal values, demonstrating good reliability of the scale (Tudoran et al., 2024). For the purposes of the present study, the analysis was limited to the instrumental value dimension with 7 items (e.g., I advocate for the rights of others, at the risk of being ridiculed or criticized), and the social terminal value dimension with 5 items (e.g., I am confident that I can make a significant contribution to society through my activities) of the original scale (Tudoran et al., 2024). Each item was rated on a five-point scale, with values ranging from 1 (strongly disagree) to 5 (strongly agree). The Cronbach α values obtained for the present study were 0.897 for instrumental values and 0.879 for social terminal values.

The level of instrumental religiosity of the respondents was determined using the Social and Individual Religiosity Scale developed by Van Camp et al. (2016). This scale assesses two dimensions of religious identity and experience—individual/social identity and intrinsic/extrinsic motivation or benefit—and consists of four subscales with 25 items: individual intrinsic religiosity, social intrinsic religiosity, personal benefits of religiosity, and social benefits of religiosity. Only the last two subscales were used in this study: personal extrinsic religiosity with 4 items (e.g., “I am religious mainly because it is a source of comfort for me”) and social extrinsic religiosity with 4 items (e.g., “It is important to me that my beliefs are compatible with those of my family and friends”). Each item was rated on a five-point scale, with values ranging from 1 (strong disagreement) to 5 (strong agreement). The Cronbach α values obtained for the present study were 0.893 for personal instrumental (extrinsic) religiosity and 0.888 for social religiosity.

The respondents’ level of interpersonal empathy was determined using the Empathy Assessment Scale, which was developed by Malakcioglu (2022) to assess empathy from a functional perspective. From the 25-item scale and three subscales, only two were selected for this study: social interaction with 4 items (e.g., “Someone else’s happiness makes me feel happy too”) and cognitive behavior with 5 items (e.g., “I understand people’s feelings from their behavior”). Each item was rated on a five-point scale, with values ranging from 1 (strong disagreement) to 5 (strong agreement). The Cronbach α values obtained for the present study were 0.824 for social interaction and 0.873 for cognitive behavior.

### 2.4. Data Analysis Methods

The data was analyzed and interpreted using IBM’s Statistical Package for the Social Sciences (SPSS) 21 and Analysis of Moment Structures (AMOS) 22. Covaraince-based SEM multivariate analysis was used to assess the relationship between the components of instrumental religiosity, the components of interpersonal empathy, and the social justice values, based to the two-stage methodology proposed by Hair et al. (2010).

The first stage consists of constructing and validating the measurement model using factor analysis. Confirmatory factor analysis (CFA) was performed using the Maximum likelihood method to confirm the factor structure of the model, which included all the study’s dependent and independent variables. To establish the scale of the latent construct and ensure model identification, the factor loading of one item was fixed at 1. The model fit was evaluated using multiple goodness-of-fit indices, including the Chi-square statistic (χ^2^), the Comparative Fit Index (CFI), the Probability of Close Fit (PCLOSE), the Root Mean Square Error of Approximation (RMSEA) and the Standardized Root Mean Square Residual (SRMR). The contribution of each observed variable to its respective latent construct was assessed by examining standardized factor loadings, with values greater than 0.50 being considered acceptable. Convergent and discriminant validity were assessed using average variance extracted (AVE), maximum shared variance (MSV), composite reliability (CR), and maximum reliability H (MaxR(H)). Convergent validity is respected if the standardized factor loading for each item evaluated is greater than 0.7, the AVE values are greater than 0.5, and the CR values are greater than or equal to 0.7. Discriminant validity is respected if the AVE square root values obtained for each factor are greater than the correlation values between factors, the MaxR(H) value is greater than the CR value, and the MSV values are less than the AVE square root values (Alrawad et al., 2023; Uslu & Ergün, 2021). Nomological validity is achieved when the correlation between the measured constructs is significant. Common Method Bias (CMB) indicates that the variance observed between the constructs of the model could be the result of the data collection method rather than the correlation between them (Podsakoff et al., 2003). This was assessed using Harman’s single-factor testing method by performing an EFA in which the loading of all items is restricted to a single factor without rotation. The absence of CMB is confirmed if the variation explained by this factor is less than 50% (Harman, 1976).

The second stage involves testing the hypotheses characteristic of the proposed model using multivariate analysis with structural equation modeling (SEM). Total effects, direct effects, and indirect effects were estimated by constructing bootstrap confidence intervals (CIs) around the estimates to assess the effects of mediators (Hair et al., 2010; Schoeps et al., 2020).

## 3. Results

### 3.1. Assessment of the Measurement Model

The measurement model, including all six variables in this study, was tested for validity (Hair et al., 2010). The normality assessments revealed that all latent factor indicators exhibited normal distributions, with the kurtosis and skewness values being smaller than two (Fidell & Tabachnick, 2003). Confirmatory analysis revealed good fit indices (Table A1): χ^2^ = 675.841, df = 362, χ^2^/df = 1.867, RMSEA = 0.045, PCLOSE = 0.9925, CFI = 0.957, and SRMR = 0.047. Common method bias was evaluated using Hardman’s single-factor test. The value of 40.42% obtained for the variance explained by a single factor is lower than the threshold value of 50%, which suggests that there is no bias in the data.

The AVE values obtained for each factor were greater than 0.50, suggesting that there is strong evidence for convergent validity and indicating that the factors explain more than half of the variation in the respective indicators (Hair et al., 2010). The CR values obtained for each factor were greater than 0.70, indicating strong internal consistency (Malhotra & Dash, 2011). All of the obtained √AVE values were greater than the correlation values between factors, indicating good discriminant validity of the model. The MSV is less than the square root of the AVE for each factor, suggesting that the variation in each construct is best explained by the items that comprise it. The MaxR(H) value is greater than the CR value for all variables except for personal religiosity, for which the two values are equal (Uslu & Ergün, 2021), suggesting that there is strong evidence for discriminant validity. The results are presented in Table 2. The values presented in Column 4 and subsequent columns correspond to the Pearson r correlation coefficients between the variables. The bold values from this section diagonal represent the square root of the AVE for each variable.

To verify the multivariate assumptions, model-estimated values were first created for all factors in the model. After examining the associated variable inflation factors (VIFs), no values greater than five were observed, indicating that there were no multicollinearity issues (James et al., 2017). Subsequently, the existence of multivariate influence points was verified by studying Cook’s distances, and no values greater than 0.07 were observed, indicating that there were no such points.

### 3.2. Assessment of the Structural Model

In order to assess the validity of the postulated hypotheses, structural equation models were constructed, with the instrumental and terminal values assigned as dependent variables and the components of instrumental religiosity and interpersonal empathy assigned as independent variables.

#### 3.2.1. Instrumental Values

The results of testing the structural model for instrumental values (corresponding to Hypothesis 1a) indicated an acceptable fit, with the following values obtained for the fit indices: χ^2^ = 460.118, df = 242, χ^2^/df = 1.901, GFI = 0.921, AGFI = 0.891, TLI = 0.958, CFI = 0.963, RMSEA = 0.046, and SRMR = 0.0433.

The CB-SEM analysis (Table A2(1)) showed that both the components of instrumental religiosity and interpersonal empathy have a direct positive effect on instrumental values and explain 53.7% of their variance, suggesting that the predictors included in the analysis are effective in identifying the factors that contribute to the respondents’ degree of adherence to social justice values. The results showed that cognitive behavior has the greatest nominal effect (β = 0.285, *p* < 0.001), followed by social religiosity (β = 0.244, *p* < 0.001), while personal religiosity (β = 0.198, *p* = 0.06) and social interaction (β = 0.172, *p* = 0.011) have less influence on the adherence to these values.

To determine whether social interaction and cognitive behavior specific to interpersonal empathy mediate the relationship between the personal and social components specific to instrumental religiosity and the instrumental values of social justice (Hypothesis 1b), two successive models were estimated. In the initial model, total mediation is proposed, with the effects of personal and social religiosity on instrumental values being only indirect achieved through social interaction and cognitive behavior. The second model proposes partial mediation, with additional direct effects specified between personal and social religiosity (exogenous factors) and the instrumental values of social justice (final outcome factors). The fit indices obtained for the total mediation model showed an acceptable fit: χ^2^ = 559.663, df = 245, χ^2^/df = 2.284, RMSEA = 0.055, GFI = 0.891, AGFI = 0.867, TLI = 0.940, CFI = 0.947, SRMR = 0.0696, and R^2^ = 0.465. For the second model, with partial mediation, the indices obtained fit the data better according to the literature (Cheung & Rensvold, 2002; Chen, 2007): χ^2^ = 513.062, df = 243, χ^2^/df = 2.111, RMSEA = 0.051, GFI = 0.902, AGFI = 0.879, TLI = 0.948, CFI = 0.954, SRMR = 0.0557, and R^2^ = 0.532.

The results presented in Table 3 showed that personal religiosity (β = 0.182, *p* = 0.043), social religiosity (β = 0.245, *p* = 0.001), cognitive behavior (β = 0.293, *p* = 0.001), and social interaction (β = 0.192, *p* = 0.006) have a direct effect on the degree of adherence to the instrumental values. Personal religiosity influences both cognitive behavior (β = 0.705, *p* < 0.001) and social interaction (β = 0.653, *p* < 0.001). Social religiosity does not substantially influence any of the components of interpersonal empathy (β = −0.062, *p* = 0.354; β = 0.011, *p* = 0.896 respectively). However, the two components of instrumental religiosity together explain 44.7% of the variance in cognitive behavior and 43.5% of the variance in social interaction.

The indirect effect of personal religiosity on the degree of adherence to the instrumental values mediated by cognitive behavior (β = 0.206, *p* < 0.001) and the effect mediated by social interaction (β = 0.125, *p* = 0.005) are substantial. No meaningful contributions were observed in the case of the indirect effects of social religiosity on instrumental value adherence, whether mediated by cognitive behavior (β = −0.018, *p* = 0.307) or social interaction (β = 0.001, *p* = 0.857). The total effect of personal religiosity on the level of commitment to instrumental values of social justice, resulting from the direct effect and indirect effects mediated by cognitive behavior and social interaction, is of a medium effect size. However, the mediating effects of cognitive behavior and social interaction do not contribute to the total effect of instrumental religiosity on instrumental values. Table 4 presents the direct, indirect and total effects of instrumental religiosity and interpersonal empathy on the instrumental values of social justice.

#### 3.2.2. Social Terminal Values

The results of testing the structural model for social terminal values (Hypothesis 2a) indicated an acceptable fit, with the following values obtained for the fit indices: χ^2^ = 437.954, df = 199, χ^2^/df = 2.201, GFI = 0.909, AGFI = 0.884, TLI = 0.950, CFI = 0.957, RMSEA = 0.053, and SRMR = 0.0455.

The results obtained from the CB-SEM analysis (Table A2(2)) showed that social religiosity, social interaction, and cognitive behavior have a positive effect on the terminal values of social justice, while the effect of social religiosity is not significant (β = 0.013, *p* = 0.821). However, the cumulative effect of the four variables in the model explains 54.6% of their variance, suggesting that the predictors included in the analysis are effective in identifying the factors that contribute to the adherence of terminal values by respondents. According to the data analyzed, personal religiosity (β = 0.337, *p* < 0.001) has the greatest nominal effect, followed by cognitive behavior (β = 0.296, *p* < 0.001), while social interaction (β = 0.203, *p* = 0.003) has somewhat less influence on these values.

To determine whether social interaction and cognitive behavior specific to interpersonal empathy mediate the relationship between the personal and social components specific to instrumental religiosity and the social terminal values of social justice (Hypothesis 2b), two successive models were estimated. In the initial model, total mediation is proposed, with the effects of personal and social religiosity on terminal values being only indirect, and is achieved through social interaction and cognitive behavior. In the second model, partial mediation is proposed, with additional direct effects specified between personal and social religiosity (exogenous factors) and the terminal values of social justice (final outcome factors). The fit indices obtained for the total mediation model showed an acceptable fit: χ^2^ = 516.663, df = 202, χ^2^/df = 2.556, RMSEA = 0.061, GFI = 0.894, AGFI = 0.867, TLI = 0.935, CFI = 0.943, SRMR = 0.0636, and R^2^ = 0.502. For the second model, with partial mediation, the indices obtained fit the data better according to the literature (Cheung & Rensvold, 2002; Chen, 2007): χ^2^ = 492.076, df = 200, χ^2^/df = 2.460, RMSEA = 0.059, GFI = 0.899, AGFI = 0.872, TLI = 0.939, CFI = 0.947, SRMR = 0.0598, and R^2^ = 0.538.

The obtained results (Table 4) showed that personal religiosity (β = 0.331, *p* = 0.001), cognitive behavior (β = 0.305, *p* = 0.003), and social interaction (β = 0.209, *p* < 0.001) have a direct effect on the degree of adherence to terminal values, while social religiosity does not directly influence adherence to these values (β = 0.012, *p* = 0.838). Personal religiosity strongly influences both cognitive behavior (β = 0.706, *p* < 0.001) and social interaction (β = 0.658, *p* < 0.001). Social religiosity does not influence any of the components of interpersonal empathy. However, the two components of instrumental religiosity together explain 44.7% of the variance in cognitive behavior and 43.4% of the variance in social interaction.

The indirect effect of personal religiosity on the terminal values mediated by cognitive behavior (β = 0.215, *p* < 0.001) and the one mediated by social interaction are substantial (β = 0.138, *p* = 0.002). There are no indirect effects of social religiosity on terminal values, both mediated by cognitive behavior (β = −0.019, *p* = 0.976) and social interaction (β = 0.0004, *p* = 0.288). The total effect of personal religiosity on the degree of adherence to the terminal values of social justice resulting from both the direct effect and indirect effects mediated by cognitive behavior and social interaction is strong (β = 0.684, *p* = 0.001). The total effect of instrumental religiosity on the terminal values resulting from the direct effect and indirect effects was not present (β = −0.005, *p* = 0.923). Table 4 presents the direct, indirect and total effects of instrumental religiosity and interpersonal empathy on social terminal values of social justice.

## 4. Discussion

This study analyzed the relationship between personal and social instrumental religiosity, the social interaction and cognitive behavior associated with interpersonal empathy, and instrumental and social terminal values of social justice. The results showed that there is a positive association between the components of instrumental religiosity and those of interpersonal empathy and instrumental values, thus confirming Hypothesis 1a. It was also found that social interaction and cognitive behavior only mediate the relationship between personal religiosity and instrumental values, with Hypothesis 1b being only partially confirmed. A positive association was also found between terminal social values and personal religiosity, social interaction, and cognitive behavior, with Hypothesis 2a being partially confirmed. In the case of terminal values, social interaction and cognitive behavior also only mediate the relationship between personal religiosity and social terminal values, partially confirming Hypothesis 2b.

The results obtained are consistent with those in the literature. Tariq et al. (2019) showed that extrinsic religiosity influences ethical decision-making, while Septianto et al. (2021) showed that people with high levels of extrinsic religiosity are more receptive to contextual cues associated with social acceptance, so they are more inclined to donate to social causes. Empirical research has also shown that empathy positively predicts prosocial behavior, with higher levels of empathy being associated with greater attention to the feelings and needs of others and a greater willingness to engage in prosocial behaviors (Pang et al., 2022). Other studies have shown that empathy promotes social cooperation and encourages involvement in activities associated with social change (Segal, 2011). However, Kaya et al. (2021) found only a positive correlation between personal extrinsic religiosity and altruism, while social extrinsic religiosity correlated negatively with altruism. At the same time, Lockwood et al. (2014) showed that both the affective and cognitive components of empathy are positively associated with self-reported prosocial behavior, with individuals with high levels of social empathy being more involved in social change processes and having higher levels of civic engagement (Hylton, 2018). Other studies have shown that empathy plays a partial mediating role between observed ostracism and compensatory behavior (Zou et al., 2022). It has also been found that the intrinsic and extrinsic dimensions of religiosity have both direct and indirect effects on the internalization of moral identity and its associated symbolic traits (Çavuşoĝlu et al., 2023).

Based on these considerations, in the proposed model, the first component, instrumental religiosity, represents the epistemic motive and influences the depth of information processing, with a high level of religiosity being associated with a high level of attention to the processing and integration of available information on social issues before forming an opinion. The second component, interpersonal empathy, represents the social motive and influences what type of information is relevant to that opinion, with a high level of empathy being associated with a greater attribution of relevance to information about social issues (Shteynberg et al., 2017). In the literature, it was shown that at the group level, the motivated information processing theory model explains why collective success is more likely in groups where high levels of epistemic motivation are coupled with high levels of prosocial motivation (De Dreu et al., 2008b). According to the present study’s results, although instrumental religiosity has a utilitarian function and serves one’s own interests, being a means of achieving social goals (Çavuşoĝlu et al., 2023), interpersonal empathy, as a positive affective trait, motivates individuals to connect emotionally with people whose rights have been violated and to respond actively by engaging in actions that contribute to resolving situations of social injustice (Peng et al., 2024).

The research undertaken is based on rigorous scientific methodologies, yielding results that complement current knowledge in the field of social justice. However, these results have been interpreted with a degree of caution due to the limitations of this study. One limitation is related to the fact that convenience sampling was used to collect empirical data. The participants are students, most of whom are of similar age and have similar academic interests, as they come from a single geographical region. Thus, the results of this study cannot be generalized to the general population. Future research could include a representative and diverse sample, with data from a larger number of cities in different geographical areas of the country. Also, the sensitivity power analysis was conducted using a multiple regression framework with manifest variables. Because the primary analyses relied on a structural equation model with latent constructs, the estimated minimum detectable effect (f^2^ = 0.029; R^2^ ≈ 0.028) cannot be directly transferred to the SEM parameters.

Another limitation stems from the methodology. The causal relationship between the three variables could not be assessed in depth, as this study was cross-sectional, with data collected at a single time interval. Since cross-sectional analysis is limited in terms of estimating mediating effects that accurately reflect the temporal antecedents of the variables, longitudinal analyses are needed to confirm the causal relationships between instrumental religiosity, interpersonal empathy, and social justice values to assess the extent to which respondents’ attitudes and behaviors change over time and to establish robust predictive models. Thus, future research may include quasi-experimental and qualitative studies conducted with other statistical methods to verify and improve the results obtained.

## 5. Conclusions

This study assessed the extent to which the degree of adherence to social justice values is conditioned by the influence of instrumental religiosity and interpersonal empathy components. The theory of motivated information processing was used as a theoretical basis for empirical research, starting from the principles of the motivated cognitive approach (also known as motivated reasoning), which states that people should be considered active and motivated processors of information. On the one hand, motivation, in the sense of refusing to follow the prescribed pattern of reasoning, can initiate cognitive processes and influence the type and nature of processing (Barclay et al., 2017). On the other hand, information processing can be influenced by the extent to which the message received is perceived as compatible with the pre-existing attitude toward the situation (Hahnel et al., 2020).

The results of the presents study showed that instrumental values of social justice are directly and positively influenced by all four variables and indirectly by personal religiosity through its influence on cognitive behavior and social interaction. In the case of social terminal values, social instrumental religiosity has neither a direct influence nor an indirect influence through empathic components. In general, the literature notes that, in the case of individuals with a high level of extrinsic personal religiosity, the benefits brought about by religious principles (e.g., happiness, inner satisfaction, and peace) constitute the motivation for their behavior, while the motivation for engaging in prosocial behaviors is to put the teachings of their faith into practice. In contrast, for individuals with a high level of extrinsic social religiosity, who are more concerned with social recognition, the motivation for prosocial behavior could be associated with the recognition they receive in the social group to which they wish to belong (e.g., with the aim of establishing stronger connections with people in the same congregation) (Kaya et al., 2021). At the same time, the essential role of social groups in establishing prescriptive norms is well documented in the literature (Yoder & Decety, 2022), with the impact of social forces that unite and divide groups on empathy, moral reasoning, and prosocial behavior being a topic of great interest to contemporary researchers (Decety & Cowell, 2015). Studies in this field have shown that, although people are capable of profound empathy and generosity, this capacity is not universal, and there are situations in which they can be indifferent or even insensitive to the suffering of others (Decety & Cowell, 2015). Within this context, religion can act as a crucial mechanism through which people manage to function in harmonious and coordinated units (Wilson et al., 1996; Paciotti et al., 2011). It has also been found that empathy can sometimes introduce bias into decision-making when individual well-being is prioritized over collective well-being, which can lead to a direct conflict with the principles of justice and fairness (Decety & Cowell, 2015). In this regard, the religious component can act on empathy by influencing human motivation, which will lead to the consolidation of innate prosocial tendencies through moral socialization (Ul-Haq et al., 2025).

Elucidating the mechanism by which people understand and act in the interest of social justice is very important from a practical point of view, as it can help solve the global challenges faced by contemporary society (e.g., poverty, inequality, racial prejudice, climate change, etc.). Although the literature, especially in the field of psychology, has provided a wealth of important information for understanding prosociality, it is still not possible to determine a priori who will act for the benefit of others and who will not (Ul-Haq et al., 2025). Given these considerations, the results of this study provide further evidence for the need to consider religiosity and empathy as important predictors of the adherence to social justice values. Understanding the relationships identified in this research can help academic researchers design educational programs that recognize and support the importance of both religion and empathy in creating a better society. Therefore, the observed relationship between religiosity, empathy, and social justice values indicates that cultivating empathic capacities could promote prosocial attitudes and social justice orientations among college students. Educational practices that emphasize perspective-taking, ethical reflection, and respectful engagement with diverse worldviews have the potential to foster the development of socially responsible behaviors.

## Figures and Tables

**Table 1:** The demographic characteristics of the participants (N = 422).

Variable	Description	N	%
Age	Emerging adult	308	73
	Adult	114	27
Gender	Female	360	85.3
	Male	62	14.7
Education level	Bachelor degree	302	71.6
	Master degree	120	28.4
Religious affiliation	Atheist/Agnostic	5	1.2
	Orthodox	314	74.4
	Catholic	14	3.3
	Neo-protestant	78	18.5
	Another denomination	11	2.6
Family members	Only child	130	30.8
	One brother/sister	172	40.8
	More than 2 children in the family	120	28.4

**Table 2:** Results of the convergent validity and discriminant validity analysis of the measurement model.

	CR	AVE	MSV	MaxR (H)	Pers. Rel.	Instr. Soc. Just.	Term. Soc. Just.	Soc. Rel.	Cog. Behv.	Soc. Int.
Pers. Rel.	0.893	0.676	0.436	0.893	0.822					
Instr. Soc. Just.	0.897	0.556	0.408	0.898	0.639	0.745				
Term. Soc. Just.	0.881	0.598	0.436	0.885	0.660	0.450	0.773			
Soc. Rel.	0.889	0.667	0.383	0.891	0.619	0.544	0.415	0.817		
Cog. Behv.	0.878	0.592	0.456	0.886	0.640	0.620	0.653	0.378	0.770	
Soc. Int.	0.827	0.547	0.456	0.840	0.626	0.588	0.618	0.412	0.675	0.740

Note: CR = composite reliability, AVE = average variance extracted, and MaxR(H) = maximum reliability H.

**Table 3:** Mediation results of SEM analysis in the case of instrumental values of social justice.

Effect	Structural Relationships	B	Bias-Corrected 95% CI				
			β	SE	Lower	Upper	*p*
Direct	Personal religiosity ⟶ instrumental values	0.114	0.182	0.088	0.006	0.354	0.043
	Social religiosity ⟶ instrumental values	0.145	0.245	0.062	0.126	0.372	0.001
	Cognitive behavior ⟶ instrumental values	0.280	0.293	0.067	0.159	0.420	0.001
	Social interaction ⟶ instrumental values	0.136	0.192	0.065	0.058	0.314	0.006
Indirect	Personal religiosity ⟶ cognitive behavior ⟶ instrumental values	0.206	0.129	0.033	0.073	0.203	0.000
	Personal religiosity ⟶ social interaction ⟶ instrumental values	0.125	0.078	0.028	0.028	0.138	0.005
	Social religiosity ⟶ cognitive behavior ⟶ instrumental values	−0.018	−0.011	0.013	−0.043	0.010	0.307
	Social religiosity ⟶ social interaction ⟶ instrumental values	0.002	0.001	0.010	−0.019	0.023	0.857
Total	Personal religiosity ⟶ instrumental values	0.321	0.515	0.060	0.399	0.628	0.000
	Social religiosity ⟶ instrumental values	0.135	0.228	0.064	0.101	0.353	0.001

Note: B = unstandardized coefficient; β = standardized coefficient; SE = standard error of B; *p* = statistical significance level of B.

**Table 4:** Mediation results of SEM analysis in the case of social terminal values of social justice.

Effect	Structural Relationships	B	Bias-Corrected 95% CI				
			β	SE	Lower	Upper	*p*
Direct	Personal religiosity ⟶ social terminal values	0.232	0.331	0.082	0.168	0.490	0.001
	Social religiosity ⟶ social terminal values	0.008	0.012	0.064	−0.118	0.136	0.838
	Cognitive behavior ⟶ social terminal values	0.326	0.305	0.073	0.081	0.449	0.003
	Social interaction ⟶ social terminal values	0.164	0.209	0.065	0.163	0.335	0.000
Indirect	Personal religiosity ⟶ cognitive behavior ⟶ social terminal values	0.215	0.150	0.039	0.082	0.236	0.000
	Personal religiosity ⟶ social interaction ⟶ social terminal values	0.138	0.096	0.032	0.039	0.165	0.002
	Social religiosity ⟶ cognitive behavior ⟶ social terminal values	−0.019	−0.013	0.012	−0.023	0.025	0.976
	Social religiosity ⟶ social interaction ⟶ social terminal values	0.0004	0.0002	0.015	−0.049	0.012	0.288
Total	Personal religiosity ⟶ social terminal values	0.479	0.684	0.059	0.564	0.797	0.001
	Social religiosity ⟶ social terminal values	−0.005	−0.007	0.070	−0.150	0.127	0.923

Note: B = unstandardized coefficient; β = standardized coefficient; SE = standard error of B; *p* = statistical significance level of B.

## Data Availability

The data supporting the findings of this study can be obtained at https://osf.io/ucjtn/overview?view_only=ad5fa2967e704c75bfb897567778bf35, accessed on 1 February 2026.

## Appendix A

### Appendix A.1. Measurement Model

**Table A1:** Results of the confirmatory analysis of the measurement model.

Fit Indices	Recommended Values	Values of Social Justice Model
χ^2^/df	≤3.0	1.867
GFI	≥0.85	0.895
RMSEA	≤0.10	0.045
PCLOSE	≥0.05	0.925
SRMR	≤0.08	0.047
CFI	≥0.90	0.957
TLI	≥0.90	0.952
IFI	≥0.90	0.957
NFI	≥0.90	0.912
RFI	≥0.90	0.902
PNFI	≥0.50	0.813
PCFI	≥0.50	0.853

### Appendix A.2. Measurement Model

**Table A2:** **1.** The coefficients of direct relationships in the structural model representing the relationship between the components of instrumental religiosity and those of interpersonal empathy, as well as the instrumental values associated with social justice. **2.** The coefficients of direct relationships in the structural model representing the relationship between the components of instrumental religiosity and those of interpersonal empathy, as well as the social terminal values associated with social justice.

**1.**						
**Structural Relationships**	**B**	**β**	**SE**	**C.R.**	***p***	**R^2^**
Personal religiosity ⟶ Instrumental values	0.124	0.198	0.045	2.731	0.06	0.537
Social religiosity ⟶ Instrumental values	0.146	0.244	0.034	4.164	<0.001	
Social interaction ⟶ Instrumental values	0.120	0.172	0.047	2.558	0.011	
Cognitive behavior ⟶ Instrumental values	0.275	0.285	0.066	4.341	<0.001	
**2.**						
**Structural Relationships**	**B**	**β**	**SE**	**C.R.**	***p***	**R^2^**
Personal religiosity ⟶ Social terminal values	0.236	0.337	0.052	4.513	<0.001	0.546
Social religiosity ⟶ Social terminal values	0.008	0.013	0.037	0.227	0.821	
Cognitive behavior ⟶ Social terminal values	0.321	0.296	0.075	4.258	<0.001	
Social interaction ⟶ Social terminal values	0.159	0.203	0.053	2.992	0.003	

Note: B = unstandardized coefficient; β = standardized coefficient; SE = standard error of B; CR = critical ratio; *p* = statistical significance level of B.
